# Intraocular Pressure Rise Linked to Silicone Oil in Retinal Surgery: A Review

**DOI:** 10.3390/vision4030036

**Published:** 2020-08-13

**Authors:** Michele Nicolai, Nicola Lassandro, Alessandro Franceschi, Alessandro Rosati, Serena De Turris, Paolo Pelliccioni, Vittorio Pirani, Cesare Mariotti

**Affiliations:** Eye Clinic, Polytechnic University of Marche, 60126 Ancona, Italy; michele.nicolai@hotmail.it (M.N.); lassandro.nicola@gmail.com (N.L.); alessandro.rosati8@gmail.com (A.R.); serena_deturris@hotmail.it (S.D.T.); paopel@hotmail.it (P.P.); piranivittorio@virgilio.it (V.P.); mariottiocul@gmail.com (C.M.)

**Keywords:** silicone oil, refractory glaucoma, emulsification

## Abstract

Silicone oil represents the main choice for intraocular tamponade in cases of complicated retinal detachment surgery. The intraocular pressure of an eye filled with silicone oil could increase, driven by a variety of different forces, according to several mechanisms. Two main conditions have been highlighted, depending on the onset: early hypertension or late glaucoma. The different types of silicone oils and their physico-chemical properties are varied and may play a role in the determination of intraocular pressure rise. The current body of literature allows for the illustration and categorization of the incidence and risk factors, as well as the pathogenesis and the management of the early postoperative hypertension subtended by an open- and closed-angle, along with the late onset silicone oil-induced glaucoma. Understanding the leading actors on the stage of ocular pressure elevation concurrently with silicone oil application for retinal surgery could help in guiding the timely and appropriate course of treatment.

## 1. Introduction

Polydimethylsiloxane (PDMS) is a linear synthetic polymer composed of repeating Si-O (siloxane) units and is chemically similar to silicone rubber, with the difference being that silicone oil (SO) polymer chains are not crosslinked and are shorter [1,2]. Silicone is an artificial synthetic substance made of the chemical element silicon (Si) (no terminal “e”), the 14th element of the periodic table and the rifest element on Earth’s crust after oxygen. SO is the liquid form of silicone, the same material that, in its solid form, is used for scleral buckling elements. SO has a specific gravity of 0.97 g/cm^3^ that is lower than water; therefore, it floats in aqueous solution when used as a vitreous substitute [3,4].

In vitreoretinal surgery, the term tamponade stands for the use of an agent to provide superficial tension on retinal breaks and seal them, preventing a relapse of retinal detachment. The most widely used internal tamponade agents are air, gas, balanced saline solution, and silicone oil, according to the need of a temporary or persistent sealing. Silicone application displaces the retina towards the eyewall via its surface tension and volume displacement [5].

SO was first introduced in vitreoretinal surgery by Cibis et al. [5,6] in 1962, providing internal tamponade by intravitreal injection [7]. It was not commonly used until the late 1970s, when Scott [8] and Zivojnovic and colleagues [9,10,11,12] promoted its use. According to Scott’s technique, SO could dissect membranes and work against traction as a stand-alone therapeutic agent without vitrectomy [13]. Its use regained popularity later in the 1980s with the advent of pars plana vitrectomy, and it was approved by the U.S. Food and Drug Administration for intraocular use in 1997 [14].

Due to its high interfacial tension (35 mN/m against water), SO covers retinal breaks and prevents transition of vitreous fluid into the subretinal space through retinal defects. Moreover, SO limits free spread of proliferative cells and biochemical mediators through the vitreous cavity, acting as a space filler. In eyes affected by proliferative diabetic retinopathy, it also has a hemostatic effect [15,16]. Another important SO advantage compared to intraocular gas is that its volume does not change over time, for this reason requiring less strict positioning and making it preferable for children or other patients unable to be optimally positioned postoperatively [17,18,19]. SO has become the gold standard tamponade in the surgery of complex retinal detachment, giant retinal tears, proliferative vitreoretinopathy (PVR), viral retinitis, and ocular trauma [20,21,22,23,24,25,26,27,28,29,30,31]. SO tamponade technique has been associated with several complications, including cataract, keratopathy, anterior chamber oil emulsification, and glaucoma [32,33,34,35,36,37,38,39]. Intraocular pressure rise may occur at any time after surgery, ranging from mild and temporary [40,41] to severe and persistent, resulting in loss of vision [2,42]. Intraocular pressure (IOP) elevation may have various pathogenetic mechanisms. We distinguish two clinical entities: an early postoperative ocular hypertension and a late onset glaucoma.

## 2. Early Post-Operative Ocular Hypertension

Early postoperative ocular hypertension (OH) after vitrectomy and SO injection can be driven by open- and closed-angle mechanisms, with an incidence ranging from 3 to 40% of cases [43,44,45,46,47,48,49,50,51,52,53]. The management of patients with acute OH after SO injection must be conducted according to the clinical setting. The pathologic mechanisms behind early IOP elevation are multiple and not yet clearly known; potential causes could be related to the exacerbation of a pre-existing undetected glaucoma [54], the severe intraocular inflammation induced by the surgery and the tamponade [38,49], the response to the postoperative steroid therapy [55,56,57], the migration of SO into the anterior chamber with consequential pupillary block, or massive infiltration of trabecular meshwork [42,50,52,58,59,60,61,62,63]. There is evidence that elevated levels of hydrogen peroxide and free radicals after vitrectomy may cause damage to trabecular meshwork cells [64,65]. Moreover, the filling of the vitreous cavity with a substance other than the vitreous could affect the structural and physiological stability of the eye [66]. The contact of SO with a trabecular meshwork already impaired from the recent surgery may trigger an inflammatory reaction leading to OH in the early phase and to an established glaucoma in the long term. SO unleashes a foreign body-like reaction in the trabecular meshwork with SO microbubbles phagocytized by macrophages [67,68,69,70,71,72]. Heavy SO (HSO) may result in a higher inflammatory reaction, probably due to its increased emulsification tendency compared to PDMS [73,74]. These etiological pathways lead to an open angle mechanism that underlies OH and is usually managed medically [2,4,37]. When the medical treatment is not sufficient to lower IOP, PDMS removal may represent the first option to consider if the risk of redetachment is low. However, in eyes with extensive synechial closure of the angle, SO removal alone hardly determines a restoration of normal IOP. In such cases, glaucoma surgery must be considered before, concomitantly, or after PDMS extraction; pupilloplasty should be considered as an early possible attempt in breaking pupillary block and reopening anterior chamber angle [75,76]. In this case, broad posterior synechiae formation represents one example of OH due to angle closure. If the angle is closed, OH may reach elevated values in a very short time. The main reason that leads to a closed angle mechanism is pupillary block [34,35,53,60,77,78,79]. Zhang et al. created an effective model for inducing sustained IOP elevation in mouse eye by intracameral injection of SO [80]. Their model demonstrated that since SO is lighter than aqueous and vitreous, it can physically occlude the pupil, preventing inflow into the anterior chamber. In this condition, the pressure in the posterior pole rises and displaces the iris anteriorly. The resulting angle closure prevents aqueous outflow through trabecular meshwork causing further IOP increase. Intraoperative PDMS overfilling could represent another cause for early OH after SO injection, but it is easily diagnosed and managed by the surgeon [81]. In human aphakic eyes, when the pressure in the vitreous cavity rises, the SO is usually displaced and it migrates towards the anterior chamber [59]. Migration of SO into the anterior chamber is a serious complication, especially in the case of heavy silicone oil because of its hydrodynamic quality. An oil droplet larger in size than the pupil into the anterior chamber can induce an acute iridolenticular block with arrest of aqueous outflow, particularly in patients lying in a supine position for a prolonged period [63]. Pupillary block leading to secondary angle closure occurs more frequently in aphakic eyes, but this complication is also described in phakic and pseudophakic eyes, probably due to zonular weakness [42,60] or iatrogenic damage due to anterior traction forces during surgery [49,62].

Immediate medical reduction of IOP with oral and topical agents is required. Face-down posturing could help aqueous humor recirculation into the anterior chamber by moving SO towards the posterior pole [82]. Patients presenting with pupillary block usually reach very high IOPs and medical treatment alone and/or face-down posturing do not tend to be effective. Definitive management to resolve pupillary block caused by SO involves inferior peripheral laser iridotomy or surgical iridectomy [83,84,85].

Careful positioning of iridotomy at the inferior limit of the SO meniscus is critical to safety and efficacy [85]. A larger pupil reduces the risk of recurrent pupil block in this context; mydriatics are suggested. A basal prophylactic iridectomy at 6 o’clock should be administered in aphakic eyes since it ensures passage of aqueous towards the anterior chamber preventing pupillary block. A superior iridectomy is usually performed when HSO is used for tamponade [61,86].

Iridectomy closure could represent a complication after the restoration of natural aqueous flow towards the anterior chamber. The mechanism for this presentation may be similar to that of the formation of posterior synechiae, which constitutes another cause for pupillary block in eyes filled with SO. In both situations, the SO bubble may serve as a scaffold for the development of a retroiridal membrane; moreover, if SO is present in the anterior chamber, it could trap iris debris, blood, and fibrin over the iridotomy site, promoting a fibrin clot formation [87]. Iridotomy reclosure is not unusual; in these cases, additional laser should be performed and the iridotomy reopened and/or enlarged. Inferior surgical iridectomies close in 11 to 32% of cases [35,49,50,61,85]. Short-term failure of laser iridotomy has been commonly described, with rates ranging from 89 to 100% [60,84]. If a long-term iridotomy failure is established, due to recurrent fibrin or inflammatory membrane formation, the usage of subconjunctival or sub-Tenon corticosteroids and intracameral tPA injections has been described as a potential remedy [87]; it could be considered as an adjunct in the treatment. If the additional laser iridectomy is found to not be effective, surgical enlargement should be promptly performed.

## 3. Late Onset Silicone Oil-Induced Glaucoma

### 3.1. Incidence and Risk Factors

In 1965, Cibis et al. [88] were the first to describe IOP increase after SO injection in retinal detachment surgery. Since then, many authors reported different percentages of incidence ranging from 20 to 50% [79,89]. Recent reports have described percentages of incidence from 3 to 30%, possibly because of the latest improvements in surgery technique and in clinical practice [2,52]. It has been estimated that 3 h after surgery mean IOP values reach 21.8 mmHg (with baseline values of 14.7 mmHg). These values remain relatively stable after 48 h with a mean of 26.0 mmHg [90]. Jabbour et al. reported post-operative increase of IOP in 48% of the eyes that underwent SO injection [91]. The authors described an elevation of IOP in 61.5% of the eyes after 1 week; in 28.7% of the eyes from 1 to 6 weeks and in 9.8% of the eyes after 6 weeks. One year after the surgery, the cumulative estimated rate of OH was 28.4 % [92]. IOP elevation after SO injection is also an important risk factor associated with best corrected visual acuity (BCVA) decrease. IOP spikes exceeding 25 mmHg have been detected in 73% of the patients reporting visual loss. It has also been described that the presence of IOP values of 21 mmHg on two consecutive visits or a value of 25 mmHg on a single observation can be considered an important risk factor causing BCVA reduction [93]. Many pre-operative and post-operative factors may lead to IOP increase. Pre-operative factors that influence IOP elevation are high myopia, diabetes, uveitis, aphakia, history of trauma, previous vitreoretinal surgery, and pre-existing glaucoma [52]. Post-operative factors include hyphema (39% of the cases), pupillary block, rubeosis iridis, anterior synechiae, extended use of post-operative topical steroids, and SO emulsification in the anterior chamber [92].

#### 3.1.1. Pre-Operative Ocular Hypertension

Pre-existing glaucoma is considered an important risk factor for IOP elevation [45,53]. In 1977, Phelps and Burton described an incidence of glaucoma before retinal detachment of nearly 7% while examining a cohort of 817 patients [94]. A recent study reported a history of preoperative glaucoma or OH (IOP ≥ 21 mmHg) of 5.9% in a cohort of 272 patients [92]. It has been described that patients with an early IOP elevation after surgery had a statistically significant higher pre-operative IOP and tended to be younger [91]. Furthermore, patients who reported IOP increase after 6 weeks had higher pre-operative IOP values than those who developed IOP elevation 1 week postoperatively. Different pathogenetic mechanisms are probably involved.

#### 3.1.2. Diabetes Mellitus

The role of diabetes in IOP elevation after SO injection has been studied extensively but is still controversial. DeCorral et al. showed that IOP elevation secondary to SO injection is independent from systemic conditions such as diabetes mellitus [95]. Other authors suggest that patients with a history of diabetes had a lower risk of developing post-operative IOP increase compared with non-diabetic patients [91]. Conversely, other authors found a significant association between diabetes mellitus and SO glaucoma [50,52,79]. It has been reported that diabetic patients with isolated proliferative diabetic retinopathy detachments had a higher risk of IOP elevation than those with isolated proliferative vitreoretinopathy detachments [53]. Moreover, Framme et al. [90] supported the hypothesis that in eyes with systemic vascular diseases (such as diabetes) IOP elevation could damage an already compromised optic nerve, resulting in visual function loss.

#### 3.1.3. Lens Status

It has been observed that patients with previous cataract surgery were more prone to develop IOP increase after 6 weeks from the surgery and had more SO drops in the anterior chamber [91]. A long-term study reported an incidence of late-onset open-angle hypertension of 15% in non-phakic eyes versus 1.4% in phakic eyes after PPV [96]. Accordingly, another study reported an incidence of late-onset open-angle glaucoma of 13% in pseudophakic eyes and 2% in phakic eyes [97]. Aphakia represents a strong risk factor for open-angle glaucoma after SO injection, increasing the risk by 10 times [50]. The Silicone Study reported that all the eyes with IOP elevation were aphakic. The reason could be the fact that the lens prevents the passage of SO into the anterior chamber. In fact, a bigger quantity of SO emulsification was found in aphakic eyes, whereas it was small or non-existent in phakic eyes [98]. However, a long-term follow-up study indicated that the presence of the lens does not prevent the development of glaucoma [36]. It was reported that over time emulsified foamy silicone globules can penetrate the protective lens barrier in many eyes, inducing IOP elevation.

#### 3.1.4. Silicone Oil in the Anterior Chamber

The presence of SO in the anterior chamber may contribute significantly to IOP increase [21,50]. Some authors reported that 43% of patients with glaucoma secondary to SO injection had SO bubbles in the anterior chamber angle [11,99]. Leaver et al. [11] reported an incidence of SO emulsification in the anterior chamber after endotamponade injection in 0.7% of patients. It has been hypothesized that SO bubbles mechanically obstruct the trabecular meshwork, blocking the outflow pathway [37]. However, despite the presence of SO emulsification in the anterior chamber or in the angle, normal IOPs have been documented [33].

Other studies suggest little or no relationships between emulsified anterior chamber SO and glaucoma [35,37,38,52]. According to the authors, IOP elevation is probably unrelated with the presence of SO emulsification but could be secondary to other pathogenetic mechanisms.

#### 3.1.5. Ocular Inflammation

Ocular inflammation is one of the factors involved in IOP elevation during silicone oil endotamponade [74]. Although in some studies inflammation rate seems to be proportional to the duration of tamponade application [4,74,100], evidence suggests that this reaction may likely persist after SO removal and may contribute to the IOP rise [101,102]. Percentages of ocular inflammation described in the literature range from 3 to 41% within 4 months from silicone oil injection [103].

Interestingly, a low incidence of IOP elevation has been described in patients with cytomegalovirus (CMV) retinitis after SO injection [104]. A study reported an incidence of 0% at 6 months and 5.9% at 1 year; however, all the patients with CMV retinitis observed were positive for the human immunodeficiency virus (HIV) [105]. It has been reported that HIV patients have lower pre-operative IOP than non-HIV patients, with a lower IOP rise on the first post-operative day [53]. The possible explanation is the diminished inflammatory response due to the compromised immune system of HIV-positive patients [104].

#### 3.1.6. Physico-Chemical Properties of Silicone Oil

Different kinds of SO are currently available for vitreoretinal surgery. They can be classified in “lighter than water” and “heavier than water” tamponade agents [15]. Standard SO, having lower density than water, provides a good support for the superior retina. On the contrary, HSO, which has a heavier density than water, provides an effective postoperative tamponade of the inferior quadrants [106,107,108,109,110,111,112,113]. Conventional SO has a specific density of about 0.97–0.98 g/mL. This “lower than water” gravity can lead to fluid accumulation in the inferior quadrants underneath the SO bubble. Furthermore, aqueous represents a pro-inflammatory milieu that is responsible for the development of proliferative vitreoretinopathy (PVR). Histopathologically, these contractile membranes are triggered by retinal pigment epithelial (RPE) cells, which scatter into the vitreous through the retinal tears, or during detachment surgery [114,115,116,117]. In 2003, the first HSO was introduced into the market as a tamponade aimed at the surgical treatment of inferior parts of the retina [118,119,120]. An important role of HSO is to displace the “PVR soup” away from the area of inferior tamponade [121].

Another important difference within the family of conventional SO is the physical parameter “viscosity”. SO viscosity ranges from 1000 cSt to 5000 cSt. Higher viscosity SO is more resistant to deformation and is thus less likely to disperse and eventually emulsify. Likewise, higher molecular weight SO, with its higher viscosity, has also been shown to be more resistant to emulsification [122,123,124]. In addition, new SO based on high molecular weight silicone chains, resulting in an overall viscosity of 2000 cSt, are widely used. They combine the advantage of the lower risk of emulsification with the ease of injection and removal, especially when using minimally invasive instruments [15]. Among commercially available HSO licensed for surgery, the mainly used products are Oxane HD and Densiron 68. Oxane HD (Bausch and Lomb, Toulouse, France) is a stable, transparent, homogenous mixture of 89% ultra-purified SO (Oxane 5700) and 11% RMN3, a partially fluorinated and hydrocarbonated olefin, with a density of 1.02 g/cm^3^ and a viscosity of 3300 cSt at 22 °C. Densiron 68 (Fluoron company, Neu-Ulm, Germany) is a stable, transparent, homogenous mixture of 30.5% perfluorohexyloctane (F_6_H_8_, a clear, homogenous semifluorinated alkane initially introduced as a SO solvent to remove SO [125,126]) and 69.5% polydimethylsiloxane (5000 cSt SO). Densiron 68 has been designed to take advantage of the high specific gravity of F_6_H_8_ and the high viscosity of SO. The resulting solution has a density of 1.06 g/cm^3^ (higher than water) and a viscosity of 1387 mPas (substantially higher than F_6_H_8_) at 25 °C. Densiron Xtra has been recently produced, with a density of 1.06 g/cm^3^ and a viscosity of approximately 1350 cSt. Its chemical structure allows easy injection, especially with 25-gauge systems. It also allows easy removal and has a low emulsification rate [127,128]. HSO has been considered to have a higher risk factor of postoperative OH than standard SO [129,130]. For this reason, an increase in IOP is a common complication after pars plana vitrectomy and HSO tamponade. Transient OH has been recorded in 13–35% of cases [63,131,132,133]. Wolf et al. [113] showed that postoperative IOP raised over 30 mmHg in 4 (12%) of 33 eyes treated with Oxane HD. In a pilot study by Tognetto and co-workers [134], the authors found an early postoperative OH in eight patients (30.7%) treated with HSO, which was easily controlled using topical or systemic antiglaucoma medications. In another pilot study, involving 42 cases recruited between Rotterdam and Liverpool, Densiron 68 was used in patients with retinal detachment. At 1 week and 1 month after HSO injection, six patients (14%) had raised IOP, and at 3 months after oil removal, three patients (7%) had IOP higher than 30 mmHg [135]. Moreover Wong et al. found that the proportion of patients with IOP of 30 mmHg or more on day 1 after the surgery was 12.7% (9 of 71 eyes) for Densiron 68 and 3.5% (2 of 54 eyes) for conventional SO. At 4 weeks, IOP of more than 30 mmHg was seen in 9 (12.7%) out of 71 eyes in the Densiron 68-treated group and 1 (1.8%) out of 54 eyes in the conventional SO group [136]. This phenomenon was due to the presence of the semifluorinated alkane that should make the compound more unstable and prone to induce emulsification and increase IOP. The presence of emulsified droplets is responsible for inflammatory reaction due to foreign body response to the emulsification [73,129,130]. All the above-mentioned heavy tamponades’ physical and molecular characteristics represent a crucial risk factor for postoperative OH, due to mechanical and inflammatory processes related to emulsification in droplets of the oil. According to various authors, emulsification occurs earlier with HSO (Oxane HD-Bausch and Lomb, Toulouse, France, and Densiron-Fluoron company, Neu-Ulm, Germany) than with standard SO [137,138,139]. The latest reports demonstrated the presence of HSO emulsification in 32.5–42.2% of eyes [36,134].

#### 3.1.7. Duration of Silicone Oil Tamponade

Many studies report that IOP increase is not influenced by the duration of SO tamponade [79,93,140]. On the other side, there are studies reporting that a long duration of tamponade represents an important risk factor for vision loss [93,141]. A profound visual loss has been documented in patients with rhegmatogenous retinal detachment without macular involvement treated by vitrectomy and SO endotamponade. It has been reported that patients with good pre-operative BCVA reported visual loss during SO endotamponade or after SO removal without any apparent explanation [141,142,143]. Christensen and la Cour [144] compared visual outcomes of patients undergoing vitrectomy for macula-on retinal detachment with SO or gas endotamponade; they found unexplained visual loss only after SO use, with an incidence of 30%. The duration of SO tamponade was the only statistically significant factor related to visual loss reported [141]. According to some authors, SO emulsification plays an important role in IOP rise after retinal detachment surgery but is unrelated to the duration of SO endotamponade [79]. The beginning of SO emulsification reported in some studies ranges from 4.3 to 5 months [35,101]. However, as long as the SO endotamponade is in place, patients with SO emulsification show slightly higher IOP values than patients without emulsification [79]. Nowack et al. [145] described IOP normalization after SO emulsification removal from the eye, unless other complications such as iris neovascularization or angle-closure glaucoma were present.

#### 3.1.8. Other Surgical Factors

Ocular hypotony or chronically elevated IOP are well-recognized complications in the use of SO for the tamponade of complicated retinal detachments with PVR [11]. It was reported that patients with PVR presented mean post-operative IOP values of more than 40 mmHg [90]. These values were found also in patients that underwent intra-operatory laser for PVR treatment, suggesting that this treatment could also have an impact on the postoperative IOP increase. Conversely, membrane segmentation is associated with a lower risk of IOP increase [91]. Other studies report an earlier SO emulsification after wide relaxing retinotomies. In this case emulsification could begin after a period of 2 months with a reduction of its tamponade effect [146]. Moreover, substances such as oligosiloxane have been identified as one of the most frequent causes of oil emulsification. Oligosiloxane has been considered an emulsification inducer since 1999 [147] and it was found in 40% of the emulsified SO explanted samples in concentrations up to seven times higher than factory concentrations [148]. Possible sources for these contaminations are tubing sets used for the transfer of SO into the eye and instruments. Remnants of SO from previous surgeries may be repeatedly in contact with cleaning substances and sterilizations temperatures. This may cause the break of the end of the long chains of poly-dimethyl-siloxane with resulting oligosiloxane formation. These oligosiloxane remnants may persist in the tubes and be mobilized in patients’ vitreous cavity during the following surgical procedures.

### 3.2. Pathogenesis

There are several mechanisms responsible for intermediate and late-onset glaucoma following the use of SO during retinal surgery, including migration of SO in the anterior chamber with possible infiltration of the trabecular meshwork by emulsified SO bubbles [11,37,53,73,79,98,103,149,150]; chronic inflammation [4,67,68,69,70,71,72,74,100,101,102,151,152,153,154,155]; synechial angle closure [11,53,78,156], rubeosis iridis [50,79,95,157], and unknown preexisting open-angle glaucoma [37,50,54]. The most common mechanism for chronic postoperative increase of IOP seems to be the secondary open-angle glaucoma caused by the blockage of trabecular meshwork [73]. The causes behind the reduction of aqueous outflow are the infiltration of SO microglobules and the intraocular inflammation, both induced by the surgery and by the emulsified SO itself, which may lead to a chronic trabeculitis [103].

Emulsification denotes a process characterized by the breakdown of the integrity of the SO bubble into smaller bubbles that are no longer able to coalesce. There are many mechanisms that can lead to emulsification including SO quality [123,124], inflammation and hemorrhagic conditions [123,158], presence of shear forces, or turbulence induced by saccadic eye movements [159,160]. Chan et al. suggested that the presence of emulsification is always underestimated, as the majority of bubbles are below 2 μm in size and remain undetected by slit lamp biomicroscopy and gonioscopy [161]. This could also explain most of the cases where the raised IOP occurs without observable emulsification. However, the clinical impact on IOP of these tiny bubbles is still debated. Findings of eyes with SO emulsification in the anterior chamber without IOP elevation suggest that different etiological mechanisms may be involved [37,50,140]. The prolonged contact of SO with the trabecular meshwork may trigger a delayed and chronic type IV hypersensitivity reaction with chemotaxis of inflammatory cells, in particular macrophages [152,153]. A foreign body-like reaction with SO microbubble phagocytized by macrophages was found in various ocular tissues [67,68,69,70,71,72].

Liu et al. found [154] that the aqueous humor of patients with SO (especially those with secondary glaucoma) had increased concentration of inflammatory mediators Interleukin-17 (IL-17), Interleukin-6 (IL-6) and Tumor Necrosis Factor-α (TNF-α), confirming the hypothesis that inflammation is involved in the pathogenesis of this type of glaucoma. HSO may trigger a greater inflammatory reaction, probably due to its increased emulsification tendency compared to PDMS [73,74]. Inflammation and emulsified SO could be related in a vicious circle in which emulsified SO triggers immune response and on the other side inflammatory proteins act as surfactants and promote more emulsification [71,162,163].

### 3.3. Silicone Oil Neuropathy

Intraocular tissue migration of SO may also have a direct toxic effect and determine SO neuropathy if the optic nerve is involved [164]. Histopathological studies of enucleated SO-filled eyes and in vivo radiologic imaging show how SO vacuoles may penetrate deep through the optic nerve to the optic chiasm and cerebral ventricles [165,166,167,168,169,170]. The pathophysiology of SO optic neuropathy is still debated. SO filling can potentially block oxygen exchange between the retinal surface and the vitreous humor and inhibit effective molecular stimuli to the inner cells, causing permanent damage to the optic nerve cells [171,172].

Some authors consider that elevated IOP may be the driving force for the migration of SO from the vitreous cavity into the optic nerve in a “pseudo-Schnabel’s cavernous degeneration” mechanism [173,174].

However, some studies also hypothesized an active transport mechanism [164,175,176]. A semi-biological model demonstrated that pressure alone seems unlikely to be the only responsible factor for the passage of SO behind the lamina cribrosa into the optic nerve [176].

A 2014 review concluded that pre-existing glaucoma and optic nerve abnormalities are the main risk factors for SO direct toxic neuronal complication [177].

### 3.4. Late Onset Glaucoma Management

Medical therapy is usually the first option to reduce IOP in SO-induced glaucoma. If necessary, aqueous suppressants combined with topical anti-inflammatory agents can control IOP in SO-filled eyes and after PDMS removal in the majority of cases [50,52]. Prostaglandin analogues have demonstrated their safety and efficacy in controlling IOP in SO-induced glaucoma with no significant difference in inflammation control compared to timolol [178]. If medical therapy fails to control IOP, the first option to consider appears to be SO removal. Several authors have shown that, in the presence of SO emulsification associated with OH, SO removal improves pressure control in the majority of patients [79,145]. Early removal of SO determines a risk of recurrent retinal detachment but many reports have shown the benefit in terms of long-term IOP control [140,179]. Budenz et al., in a retrospective study of surgical intervention for secondary glaucoma after pars plana vitrectomy and silicone oil injection, found a success rate of 69%, 60%, 56%, and 48% at 6, 12, 24, and 36 months, respectively, in patients who underwent silicone oil removal alone [78]. In such cases, a careful removal of the emulsified SO is very important to reduce the inflammatory reaction. Repeated fluid–air exchange in the vitreous cavity and a complete aspiration of emulsified droplets in the anterior chamber are essential to prevent the risk of residual SO retention in the eye. If PDMS removal is infeasible due to the retinal condition, temporary measures such as laser therapy can be performed. Although there are only a few reports, selective laser trabeculoplasty (SLT) can be considered as an adjunct therapy in SO-induced glaucoma. Zhang et al. reported a success rate of 59.5% and an IOP reduction of 4.7 mmHg (20.3%) at 12 months [180]. This study lacks a control group and is limited to a small cohort; however, it shows that SLT may have a role in the management of open-angle SO-induced glaucoma. Despite this, especially in cases of extensive synechial closure, PDMS removal and medical therapy may not adequately control IOP. Therefore, glaucoma surgery appears the inevitable choice for these eyes. Surgical management of SO glaucoma should be decided individually according to visual function, gonioscopic evaluation, level of IOP elevation, and conjunctival status. Early intervention is essential, as visual loss can be very subtle in this kind of secondary glaucoma. The decision of performing glaucoma surgery must also take into account that filtering surgery in previously vitrectomized eyes can be very challenging, as it carries higher failure rate and risk of complications. Surgical intervention options to manage SO-induced glaucoma include trabeculectomy, Ex-PRESS minishunt, deep sclerectomy, glaucoma drainage devices (GDD), and trans-scleral cyclophotocoagulation [50]. There are no reports of the effectiveness of minimally invasive glaucoma procedures in the management of SO-related glaucoma. Poor long-term success of mitomycin-augmented trabeculectomy after vitreoretinal surgery has been widely reported [181,182]. El Saied et al. compared the outcome of four different surgical modalities (trabeculectomy, deep sclerectomy, Ahmed valve, Ex-PRESS minishunt) for management of persistent glaucoma after SO removal in prospective randomized trial. In this study, Ex-PRESS minishunt had the highest complete success rate [183]. Glaucoma drainage implants provided an interesting surgical option in cases of refractory SO-induced glaucoma that can be considered as a first-line treatment or after a failed previous glaucoma surgery [37,184]. However, the surgical prognosis is poorer if compared with primary glaucoma. Ishida et al. showed that the success rate of Ahmed glaucoma valve implantation in eyes treated with pars plana vitrectomy and SO endotamponade was significantly lower compared with the results of eyes that had not been treated with SO. The success rate at the last follow-up examination, with a mean follow-up of 2 years, was 70.2% in patients with SO and 87.2% in patients without SO [185]. GDD can be placed in the inferior quadrants (inferotemporal or inferonasal) in patients with SO emulsification to minimize the infiltration of SO through the tube. However, migration of SO in the tube has also been described in cases of inferior placement [186]. Tube placement in pars plana has shown similar results in efficacy and fewer complications when compared to the traditional anterior chamber placement. This approach should be preferred in cases of coexisting corneal disorders [187].

The reason for the higher failure rate of all filtering procedures appears to be the inflammatory reaction and increase of fibrosis caused by the migration of emulsified SO droplets under the conjunctiva [188,189,190]. In cases where the visual function is poor, cyclodestructive procedures have been used to reduce intraocular pressure in refractory SO-induced glaucoma. Cyclodestruction can be achieved with cyclocryotherapy or diode laser cyclophotocoagulation. Retrospective studies on cyclodiode report a good success rate but also a high rate of retreatment [191]. More than 50% of the patients require a second treatment to obtain a good IOP control at 1 year of follow-up [192]. Chronic hypotony is a feared complication after cyclodestructive procedures. Transscleral diode treatment does have a lower incidence of postoperative low pressure compared to cyclocryotherapy but titrating the laser effect remains difficult [193]. Endoscopic cyclophotocoagulation provides the advantage of delivering the laser directly to the ciliary body [194]. This technique has shown better results when compared to Ahmed drainage device in refractory glaucoma [195].

### 3.5. Future Vitreous Substitute

The main silicone oil feature that leads to the significant complications discussed in this paper is probably its hydrophobic nature. One solution might be to develop an alternative vitreous body substitute, a hydrophilic vitreous body substitute that fits the natural and complex function of a juvenile, healthy vitreous.

Cross-linked hydrogels are synthetic polymer networks that are expanded throughout their volume by water. Smart hydrogels can create a three-dimensional structure characterized by transparency, biocompatibility, and mechanical flexibility [196]. These polymers have only been used on an experimental basis and, at this time, their major disadvantage is the intravitreal inflammation due to immune system activation [197]. Hydrogels seem to be promising candidates as infill biomaterials for the treatment of retinal detachment. Recently, a new class of hydrogel, applied as an artificial vitreous body for over a year without adverse effects in the eyes of rabbits, has been reported [198], and a clinical trial has evaluated safety and tolerance of intravitreal hydrogel following vitrectomy surgery in humans [199].

## 4. Conclusions

The evolution of minimally invasive vitreo-retinal surgery has significantly reduced the incidence of SO-induced glaucoma as highlighted by the most recent reports. However, this complication remains one of the principal causes of visual loss in these eyes. Surgical treatment in refractory cases has a poor prognosis if compared with primary glaucoma.

## Methods of Literature Search

There were no specific exclusion/inclusion criteria for our literature search. Published work written in other languages were analyzed by abstract only, which is usually available in the English language. Searches included terms similar to “silicone oil”, “glaucoma”, and “ocular hypertension”. All papers found relevant to this manuscript upon review were included upon the authors’ discretion. Online applications used for the searches were PubMed database and clinicaltrials.gov.
